# Commentary: Validation of a Ramp Running Protocol for Determination of the True VO_2max_ in Mice

**DOI:** 10.3389/fphys.2017.00330

**Published:** 2017-05-24

**Authors:** Koen K. Lemaire, Rémi Thomasson, Philippe Noirez, Richard T. Jaspers, A. J. van Soest

**Affiliations:** ^1^Human Movement Sciences, Faculty of Behavioural and Movement Sciences, Amsterdam Movement Sciences, Vrije Universiteit AmsterdamAmsterdam, Netherlands; ^2^Groupe Physiologie Expérimentale, Institut de Recherche bio-Médicale et d'Epidémiologie du Sport, Université Paris DescartesParis, France; ^3^Laboratory for Myology, Human Movement Sciences, Faculty of Behavioural and Movement Sciences, Amsterdam Movement Sciences, Vrije Universiteit AmsterdamAmsterdam, Netherlands

**Keywords:** flow-through respirometry, room calorimetry, metabolism, mice, *VO*_2max_

Maximal oxygen uptake $\left(\dot{V}O_{2\max}\right)$ is an important physiological parameter, which is often used to evaluate the physiological effect of (training) interventions in mice. $\dot{V}O_{2\max}$ is typically defined as the $\dot{V}O_{2}$ reached during exhaustive exercise, where a $\dot{V}O_{2}$ plateau is observed despite an increase in workload, in combination with a respiratory exchange ratio (RER) > 1 (Ayachi et al. (2016); hereafter referred to as Ayachi2016). However, as adequately pointed out by Ayachi2016, a widely accepted standard protocol for measuring mouse $\dot{V}O_{2\max}$ is currently lacking.

To arrive at such a standard protocol Ayachi2016 have measured the peak $\dot{V}O_{2}$ (highest 15 s averaged $\dot{V}O_{2}$ during a trial, $\dot{V}O_{2\text{peak}}$) using six protocols (three treadmill velocity profiles, at two treadmill inclinations) requiring mice to run on a treadmill in an air-tight chamber (volume of air inside the chamber, *V*_ch_). Fresh air was blown into the chamber at a rate ${\dot{V}}_{\text{in}}$, circulated with a fan, and extracted and sampled for oxygen and carbon dioxide content. Based on their results, Ayachi2016 conclude that a ramp velocity protocol with an initial velocity of 3 m/min (0.05 m/s) and a constant acceleration of 3 m/min^2^ (0.0083 m/s^2^) at an inclination of 0 degrees is best suited for estimating the true $\dot{V}O_{2\max}$.

As explained elsewhere (Lighton and Halsey, 2011), the relation between the mouse $\dot{V}O_{2}$ and the measured oxygen concentration in the chamber is well described by a first order linear system with a time constant and a static amplification that are known (Bartholomew, 1981; Christensen, 1946) to equal $\frac{V_{\text{ch}}}{{\dot{V}}_{\text{in}}}$ and $\frac{1}{{\dot{V}}_{\text{in}}}$, respectively. In the experiments of Ayachi2016 we estimate that *V*_ch_ = 1,350 mL and ${\dot{V}}_{\text{in}}$ = 11 mL/s, resulting in a time constant of about 120 s. Using the latter values and the definitions for $\dot{V}O_{2}$ plateau and $\dot{V}O_{2\text{peak}}$ from Ayachi2016, we have simulated the results of the ramp 3, 0 degree protocol from Ayachi2016. The results of this simulation are shown in Figure 1. The modeled, “true,” mouse $\dot{V}O_{2}$ signal (i.e., input in our simulation, Figure 1B, blue, solid curve) was adjusted such that the “measured” signal (Figure 1B, red, dashed curve) resulted in a plateau region of 57 s and was based on the assumptions that (1) the mouse (and the measurement system) started the test in rest; (2) the steady state mouse $\dot{V}O_{2}$ increases linearly with running speed (as suggested by Ayachi2016); and (3) the mouse itself acts as a linear system with a time constant of 20 s. The resulting $\dot{V}O_{2\text{peak}}$ equaled 0.995 of the input value for $\dot{V}O_{2\max}$ confirming that input $\dot{V}O_{2\max}$ was indeed measured during the trial. However, as can be appreciated from Figure 1, in order to observe the 57 s plateau reported in Ayachi2016, the mice must have reached their true $\dot{V}O_{2\max}$ almost 6 min prior to the end to the trial. This is a surprising result, considering that the belt speed continuously increased during this 6 min period, whereas oxygen consumption remained constant at $\dot{V}O_{2\max}$.

**Figure 1:**
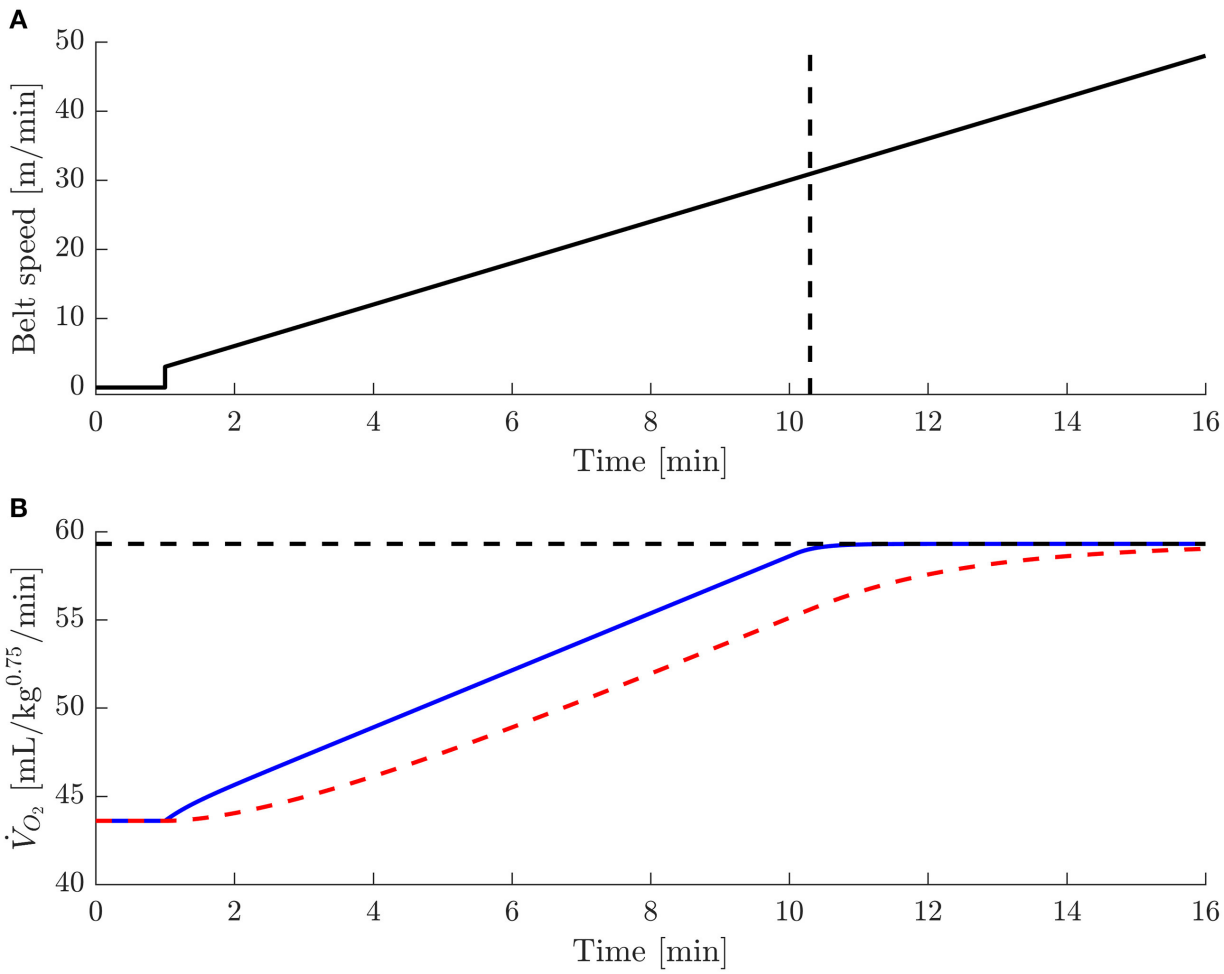
**Summary of simulation results of Ayachi2016's ramp 3, 0 degrees protocol. (A)** Belt speed against time. The vertical dashed line indicates when the “true” $\dot{V}O_{2}$ reaches the value for $\dot{V}O_{2\text{peak}}$ calculated from the “measured” $\dot{V}O_{2}$ **(B)**. **(B)** Modeled “true” $\dot{V}O_{2}$ (blue, solid curve) and modeled “measured” $\dot{V}O_{2}$ (red, dashed curve), calculated from the true $\dot{V}O_{2}$ under the assumptions detailed in the text, against time. The horizontal dashed line indicates the assumed value for $\dot{V}O_{2\max}$, which was taken from Ayachi2016.

It should be noted that our assumptions regarding the shape of the input signal represent a best case scenario; other realistic input signals (e.g., an exponential) that are parameterized to result in a comparable plateau in the measured $\dot{V}O_{2}$ signal would require the true $\dot{V}O_{2\max}$ to be reached even earlier during the trial. Moreover, although the dynamics of the measurement system are best directly measured, as suggested by Lighton and Halsey (2011), our estimation of its time constant is the theoretical minimum, and thus also constitutes the best case scenario.

As is clear from our simulation results (Figure 1), while measuring mouse $\dot{V}O_{2}$ during chamber calorimetry, the dynamics of the measurement system is a complicating factor. As discussed in (Lighton and Halsey, 2011), there are two ways to improve (i.e., decrease) the time constant of the measurement system: (1) decrease the free volume inside the chamber, and (2) increase the flow rate. Regarding option 2, we note that this leads to an undesirable decrease of the static amplification and thus to a deteriorated signal to noise ratio. Furthermore, it might compromise perfect mixing of the gasses inside the chamber, which is a prerequisite for valid measurements.

In sum, while acknowledging the importance of the issues discussed in Ayachi2016, we stress that the dynamics of the measurement system must be taken into consideration when interpreting experimental results that are obtained using chamber calorimetry. In that regard, it is important that protocols for determination of oxygen consumption in mice should be chosen such that the time constant of the particular measurement system is adequate in relation to the kinetics of oxygen uptake. One way to achieve this may be to impose similar exercise protocols in a setup in which the free volume inside the chamber is much smaller, such as the swimming setup described in Grondard et al. (2008).

## Author contributions

All authors listed, have made substantial, direct and intellectual contribution to the work, and approved it for publication.

### Conflict of interest statement

The authors declare that the research was conducted in the absence of any commercial or financial relationships that could be construed as a potential conflict of interest.

## References

[B1] Ayachi M. Niel R. Momken I. Billat V. L. Mille-Hamard L. (2016). Validation of a ramp running protocol for determination of the true VO_2max_ in mice. Front. Physiol. 7:372. 10.3389/fphys.2016.00372 27621709 PMC5002025

[B2] Bartholomew G. (1981). Instantaneous measurements of oxygen consumption during pre-flight warm-up and post-flight cooling in sphingid and saturniid moths. J. Exp. Biol. 90, 17–32.

[B3] Christensen B. G. (1946). A simple apparatus for simultaneous measurement of the standard metabolism and the respiratory quotient in small laboratory animals, supplemented with investigations on normal and hypophysectomised rats. Acta Physiol. Scand. 12, 12–26. 10.1111/j.1748-1716.1946.tb00362.x

[B4] Grondard C. Biondi O. Pariset C. Lopes P. Deforges S. Lécolle S.. (2008). Exercise-induced modulation of calcineurin activity parallels the time course of myofibre transitions. J. Cell. Physiol. 214, 126–135. 10.1002/jcp.21168 17559060

[B5] Lighton J. R. B. Halsey L. G. (2011). Flow-through respirometry applied to chamber systems: pros and cons, hints and tips. Comp. Biochem. Physiol. A Mol. Integr. Physiol. 158, 265–275. 10.1016/j.cbpa.2010.11.026 21134483

